# Optimization study of mine fire sensor based on grey correlation analysis

**DOI:** 10.1371/journal.pone.0313272

**Published:** 2025-02-11

**Authors:** Xiaokun Zhao, Minghao Ni, Wencai Wang, Jianing Wang, Hongwei Wang

**Affiliations:** 1 School of Coal Engineering, Shanxi Datong University, Datong, Shanxi, P.R. China; 2 School of Mining and Coal, Inner Mongolia University of Science and Technology, Baotou, Inner Mongolia Autonomous Region, P.R. China; SRM-RI: SRM Institute of Science and Technology (Deemed to be University) Research Kattankulathur, INDIA

## Abstract

In order to address the issues of fire alarm delay, omission, and false alarms caused by the current setup of mine fire sensors, which collect single disaster information, have fixed distribution, and relatively independent data collection, this study utilized FDS numerical simulation software and fire similarity experiments. The aim was to investigate the characteristics of fire gases, temperature, and wind speed. To optimize the number and location of fire sensors in mines, the mathematical method of grey correlation analysis was proposed. Additionally, the critical time for fire hazards to spread to other tunnels was determined by detecting the CO content of ventilation nodes in tunnels with different wind speeds. Grey correlation analysis was employed to determine the critical time for fire hazards to spread to other tunnels. The CO content of the ventilation node is measured to determine the time at which the fire hazard may spread to other tunnels. Grey correlation analysis is then used to compare and correlate the CO content of the ventilation node and the wind speed with the fire characteristic gases, wind speed, and temperature of the tunnel under different wind speeds. Additionally, taking into account the safe escape time for personnel suggested by Marchant, an optimization scheme for the sensors is proposed.

## 1 Introduction

Mine fires are a significant threat to underground operators, as the confined space and limited ventilation underground facilitate the rapid spread of smoke. This leads to the expansion of the disaster area and can also impact other connected branch tunnels [1]. The location of the mine fire is uncertain, but the distribution of underground sensors remains fixed. The collected information from these sensors is independent of each other. The alarm response mechanism is typically triggered when the collected disaster information reaches the upper limit of the sensor’s threshold [2–5]. The location of the fire source and the distance between the non-fire factors and the sensors can influence the alarm system, leading to false alarms, omissions, and delays. This can cause underground operators to miss the optimal time to escape. Additionally, the high-temperature smoke flow can spread to other tunnels, further expanding the disaster area [6].

With the advancement of information intelligence, the multi-sensor information fusion fire detection method has the capability to address the issues of delayed information collection and false alarm omission that are commonly encountered in traditional fire alarms. Yu Lingfeng [7] proposed a solution to enhance the accuracy of fire alarms by introducing an intermediate station between the sensors and the monitoring point. This intermediate station locally decodes the data collected by similar sensors, thereby ensuring that the system’s fire recognition is not reliant on a specific sensor; Hua Zhang [8] utilized numerical simulation software and neural networks to enhance the structure of the multi-data fusion technique. The study involved sampling both open and shaded fires to improve the accuracy of fire warning In related literature [9–12], a multi-sensor fire warning system for fire simulation was proposed using numerical simulation software. The results demonstrated that the incorporation of multi-factor fusion in the fire warning system could effectively reduce the false alarm rate; Jingmai Wang [13] conducted data processing using FDS numerical simulation software, considering multiple factors such as building structure, fire source type, location, detection point distance, and ventilation. A multi-sensor coupled detection model was proposed and the fire alarm response time was compared before and after the improvement. The findings indicated a reduction in the fire alarm time by 14.7%. Ishii et al. [14] developed a neural network for fire detection that incorporated three types of data: temperature, smoke, and CO concentration. This approach enhanced the resilience of the fire alarm system.

Data processing of fire characteristic parameters is a widely used approach for achieving multi-sensor information fusion in fire detection. Numerous scholars have analyzed these parameters using neural networks and related algorithms, and have proposed utilizing the regularity of fire characteristic parameters to efficiently predict and warn against fires. For instance, Okayama [15, 16] designed a neural network system that combines odor sensors and smoke detectors, and trained the network to differentiate between fire and environmental disturbances. Ilke [17] proposed a multi-data fuzzy logic algorithm that combines three characteristic parameters: CO concentration, smoke, and temperature. Full-size fire experiments were conducted to process the data from the sensors and apply them to the fuzzy logic algorithm. The results demonstrated that this algorithm could effectively provide early detection of fire. In a study by Xuegui Wang [18], the data collected from multiple sensors was analyzed. The study found that there is a variation in the prediction results of different sensor combinations, even under the same conditions. Wang also explored and investigated various processing methods for fire detection information data. In the field of data processing, previous studies [19, 20] have analyzed data using grey correlation analysis to address the issue of data coupling in the context of residual information. These studies have also demonstrated the practicality and effectiveness of the grey correlation model. While there have been abundant results in research on prediction models for fire multi-featured parameter data processing by other scholars [21–23], there is limited research on optimizing the deployment of sensors and determining the optimal number of sensors for collecting disaster information in downhole scenarios.

Due to the unique environment and spatial structure of underground spaces, the utilization of a large number of sensors in a multi-sensor information fusion fire detection platform can lead to excessive consumption of underground resources. Therefore, this study investigates the impact of mine fire characteristic gases and the throttling effect of fire on mine wind speed through similar experiments and numerical simulations. Additionally, a mathematical method based on grey correlation degree analysis is proposed to process the mine fire characteristic parameter information, optimize the number and placement of mine fire sensors, enhance the responsiveness and accuracy of the multi-parameter fire alarms, reduce false fire alarms, and optimize the resource allocation of the underground fire protection system.

## 2 Similar experiment

### 2.1 Model experimental parameters

To establish a scaled model for the section of 4m (height) × 5m (width), it is necessary to consider the principles of fluid dynamics and motion similarity. The proportions between the scaled model and the actual model can be described using the formula [24]:

$$\lambda_{l}=\frac{l_{m}}{l_{p}}$$ (1)

where *λ* is the scaling relationship between the scaled model and the full-size model, *l* is the feature length, m. *m* is the scaled model corner scale and *p* is the full-size model corner scale.

In order to account for the experimental site and equipment factors, a reduced scale model was created for the fire experiments. The scale relationship of this model was approximately 6.5, ensuring geometric similarity. The specific parameters of the model are shown in Table 1:

**Table 1:** Structure and parameters of the scale-down model.

Name	Size/cm	Material
Upper and lower bottom	80×1000	Steel
Sidewall	60×100	Reinforced glass
Frame	3.8×3.8	Square Steel Pipe
Hibachi	20×30×1.5	Aluminum

**Fig 1:**
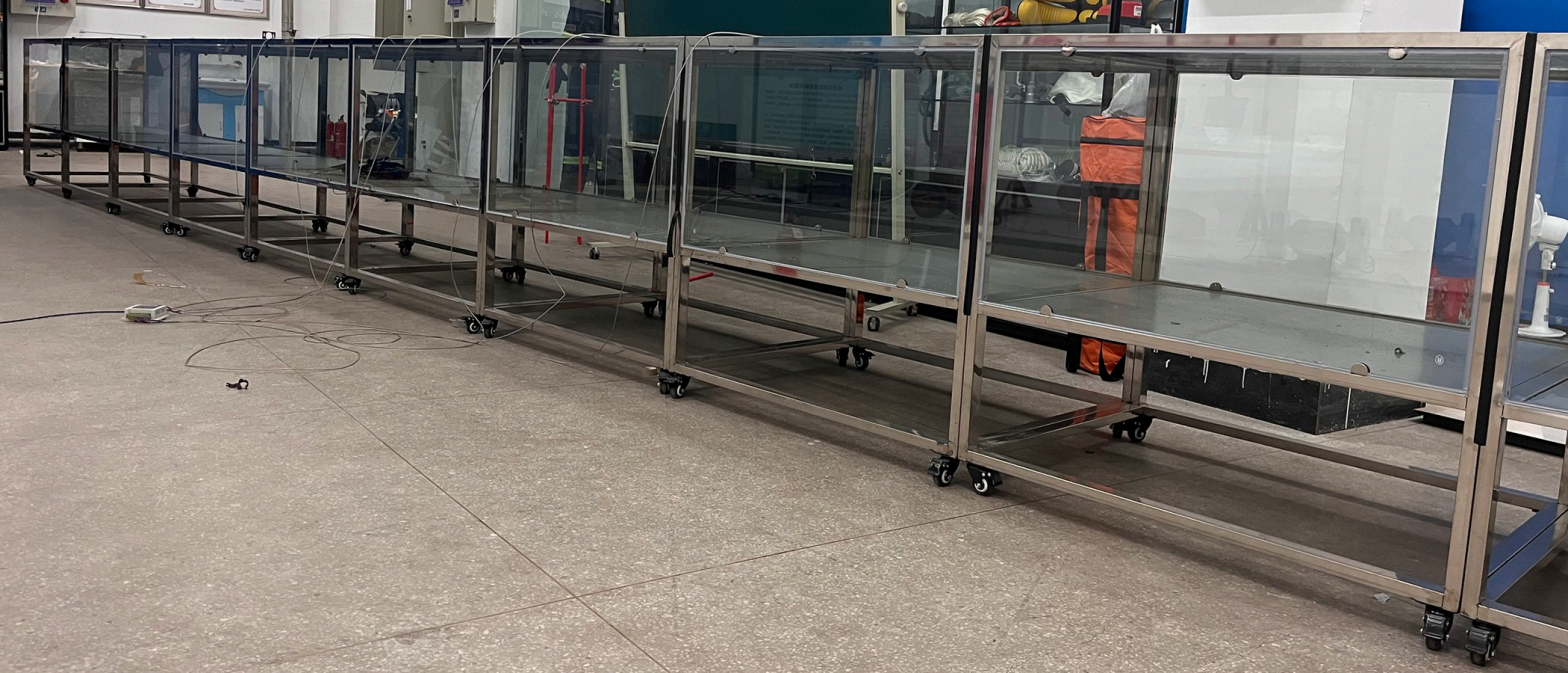
Experimental model diagram.

The ambient temperature was measured to be 25°C. To monitor temperature and CO2 concentration during a fire in the scale model, several pt100 thermocouples and gas concentration sensors were installed on the top. The fire source was positioned 200 cm away from the fan. The first sensor was placed directly above the fire source, with additional sensors positioned every 40 cm in the downwind direction. Sensors were also installed at the end section of the model. The chosen ventilation equipment consisted of a jet fan with a diameter of 48 cm. Experimental measurements determined an average wind speed of 0.8m/s. The equipment demonstrated good airtightness. The scaled model and experimental structure are illustrated in Fig 1.

Considering the flammable materials in the underground coal mine, we selected fir wood chips and fir wood blocks as ignition materials, considering their flammability. Solid alcohol was used to ignite these materials, and the fuel attribute parameters are shown in Table 2:

**Table 2:** Fuel parameters.

Fuel name	Combustion Heat	Radiant Heat Flow	Density
Cedar	11.302Mj/Kg	17.2Kw/m^2^	357.1Kg/m^3^

### 2.2 Model fire experiment

**Fig 2:**
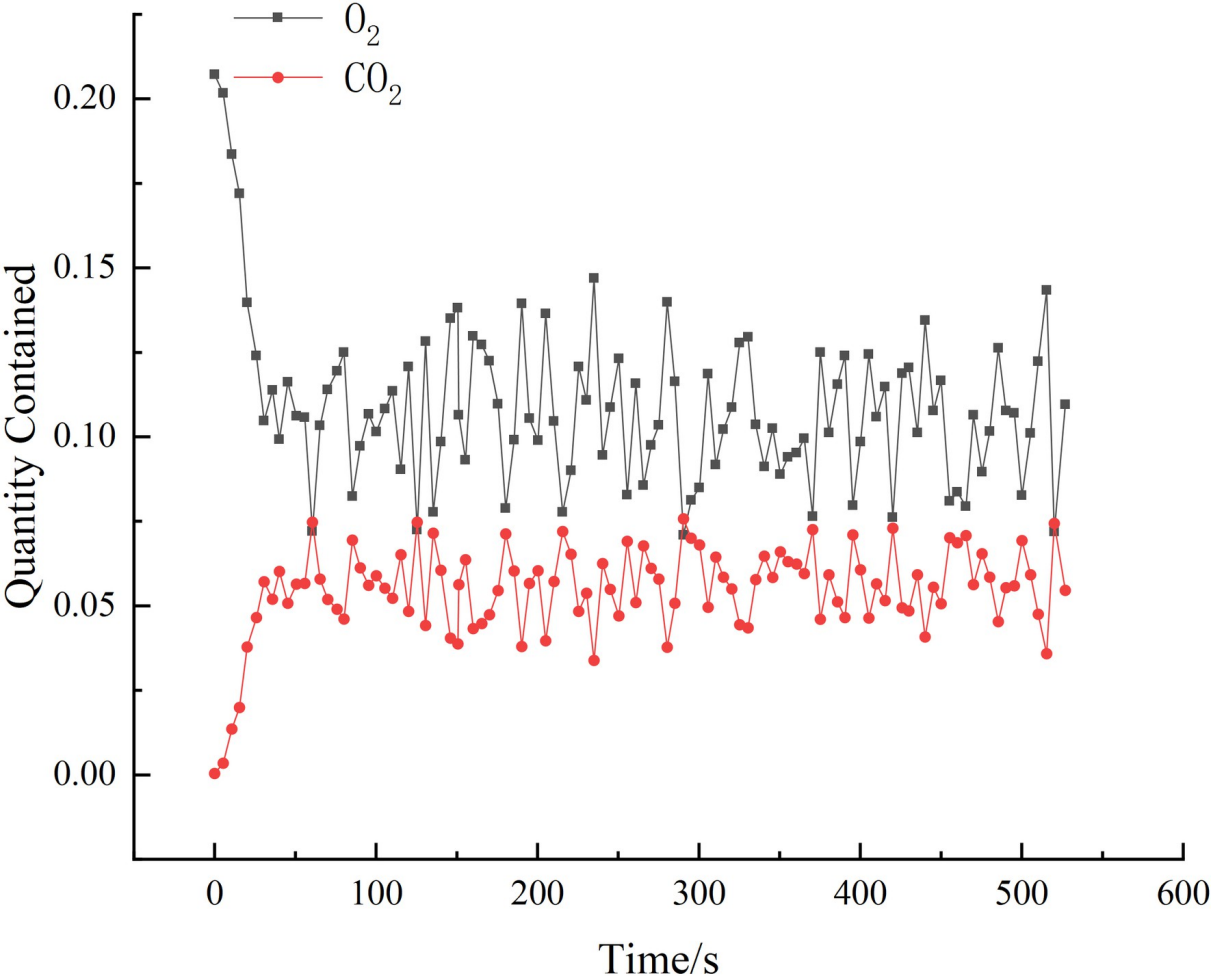
Plot of O_2_ and CO_2_ over time.

The study categorizes similar fire experiments into three stages: the preliminary stage of the fire source, the stable stage, and the fire extinguishing stage. The oxygen concentration sensor and CO2 signals are analyzed to determine the heat release rate of the combustible material and the time it takes to reach the stable stage. Fig 2 shows that the oxygen concentration reaches its lowest point and the CO2 concentration reaches its peak at approximately 61s. This indicates that the fire source reaches its maximum heat release rate around 61s.

**Fig 3:**
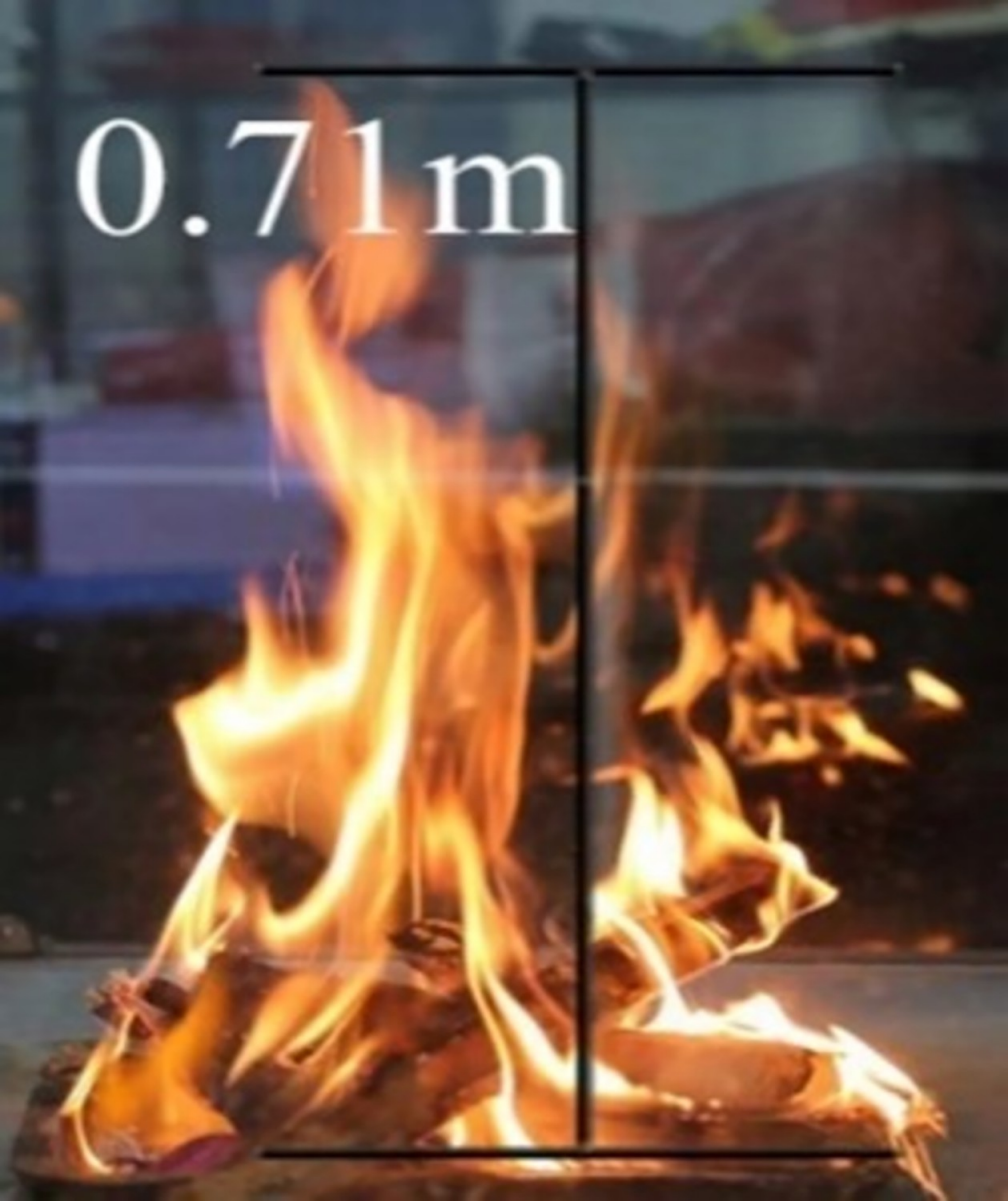
Flame height map.

By observing and measuring the flame height at 61s, which is about 0.71m, as shown in Fig 3. Heskestad [25] gave an expression for the flame height at standard conditions:

$$L=-1.02D+0.235Q^{2/5}$$ (2)

Where *L* is the flame height, m; *D* is the equivalent diameter of the fire source, m; *Q* is the heat release rate of the fire source, KW. By calculation, the equivalent diameter of the fire source is about 0.27m, and the heat release rate of the fire source is about 36.9KW from Eq (2).

Using FDS numerical simulation software, a numerical simulation model of the experimental conditions is established for fire simulation with the following boundary conditions shown in Table 3:

**Table 3:** Numerical simulation software boundary conditions.

Boundary Condition	Parametric
Wall	TITLE MATERIAL
Roof and Floor	STEEL
Fire Source Size	20cm×20cm
Reaction	Cellulose Combustion
Fire Source Power	36.9Kw

**Fig 4:**
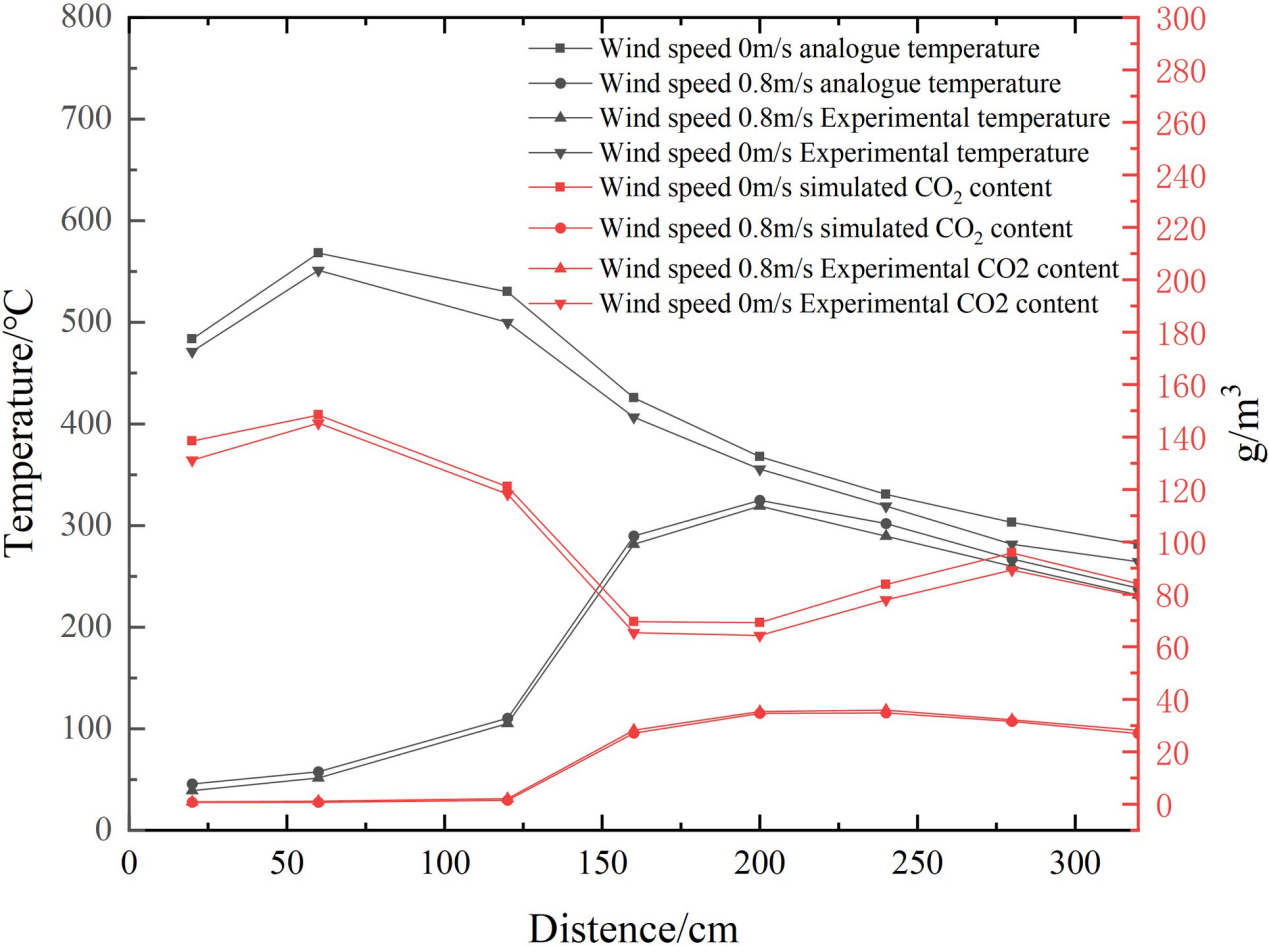
Comparison of experimental and simulated fire characteristics parameters at the 61s moment.

Numerical simulation and fire experiments were conducted in two different ventilation environments: one with no wind (0m/s) and the other with a wind speed of 0.8m/s. The experiment lasted 527 seconds and the thermocouples and CO_2_ concentration sensors at the same distance for each experiment were selected for comparison between the numerical simulation and the scaled model experiment at the 61st second. The comparison graph is shown in Fig 4.The maximum temperature error between the numerical simulation and fire experiment was found to be 4.6% in the absence of wind, and approximately 4.5% at a wind speed of 0.8 m/s. As for gas measurements, the maximum error was 3.9% and 3.4% under windy and windless conditions, respectively. These results indicate a satisfactory agreement between the numerical simulation and similar experiments under both boundary conditions.

## 3 Mathematical model for grey correlation analysis

### 3.1 Basic idea

The grey correlation analysis method is a useful tool for assessing the strength of the relationship between factors by comparing the similarity of their trends of change. This method enables us to determine the impact of sub-sequences on the parent sequence and understand the contribution of factors to the overall behavior of the system.

The grey correlation analysis method is used for dimensionless data processing and dimensionality reduction of multi-dimensional data. In this analysis, the first step is to confirm the target sequence and the comparison sequence. The basic idea is to compare the geometric shapes of these sequences and judge the degree of similarity as a measure of correlation. The closer the curves, the higher the correlation, and vice versa, indicating a lower degree of correlation.

### 3.2 Mathematical approach to grey correlation

Let the target data column *x*_*0*_, which can be written as:

$$x_{0}=\{x_{0}(1),x_{0}(2),\ldots x_{0}(n)\}$$ (3)

Let the comparison sequence be *x*_*i*_, which can be written as:

$$x_{i}=\{x_{i}(1),x_{i}(2)\ldots x_{i}(n)\}i=1,2\ldots m$$ (4)

It can also be written as an n × m order matrix:

$$\left|\begin{array}{cccc} x_{1}(1) & x_{1}(2) & \ldots & x_{1}(n)\\ x_{2}(1) & x_{2}(2) & \ldots & x_{2}(n)\\ \vdots & \vdots & \ddots & \vdots \\ x_{m}(1) & x_{m}(2) & \ldots & x_{m}(n) \end{array}\right]$$ (5)

And then the data standardization was carried out and the data was made dimensionless and its standardization was calculated as [26]:

$$x_{j}=\frac{{x_{j}}^{*}}{\left(|x_{j}-{x_{j}}^{*}|+{x_{j}}^{*}\right)}$$ (6)

Let the minimum difference between the two levels be Δmin, which is calculated as:

$$\Delta_{\min}=\underset{i}{\min}\;\underset{k}{\min}\left|x_{0}(k)-x_{i}(k\right)|$$ (7)

Let the maximum difference between the two levels be Δmax, which is calculated as:

$$\Delta_{\max}=\underset{i}{\max}\;\underset{k}{\max}|x_{0}(k)-x_{i}(k)|$$ (8)

Then the grey correlation coefficient of *x*_*i*_ to *x*_*0*_ with respect to the k index can be expressed as [26]:

$$\xi_{i}(k)=\frac{\Delta \min+\varsigma \Delta \max}{|x_{0}(k)-x_{i}(k)|+\varsigma \Delta \max}$$ (9)

where *ς* is the resolution factor, *ς*∈[0,1]. The resolution factor is usually taken as 0.5.

In grey correlation analysis, the absolute correlation of each object of i is [26]:

$$r_{i}=\sum_{k=1}^{n}W(K)\cdot \xi_{i}(K)$$ (10)

### 3.3 Application of grey correlation analysis

**Fig 5:**
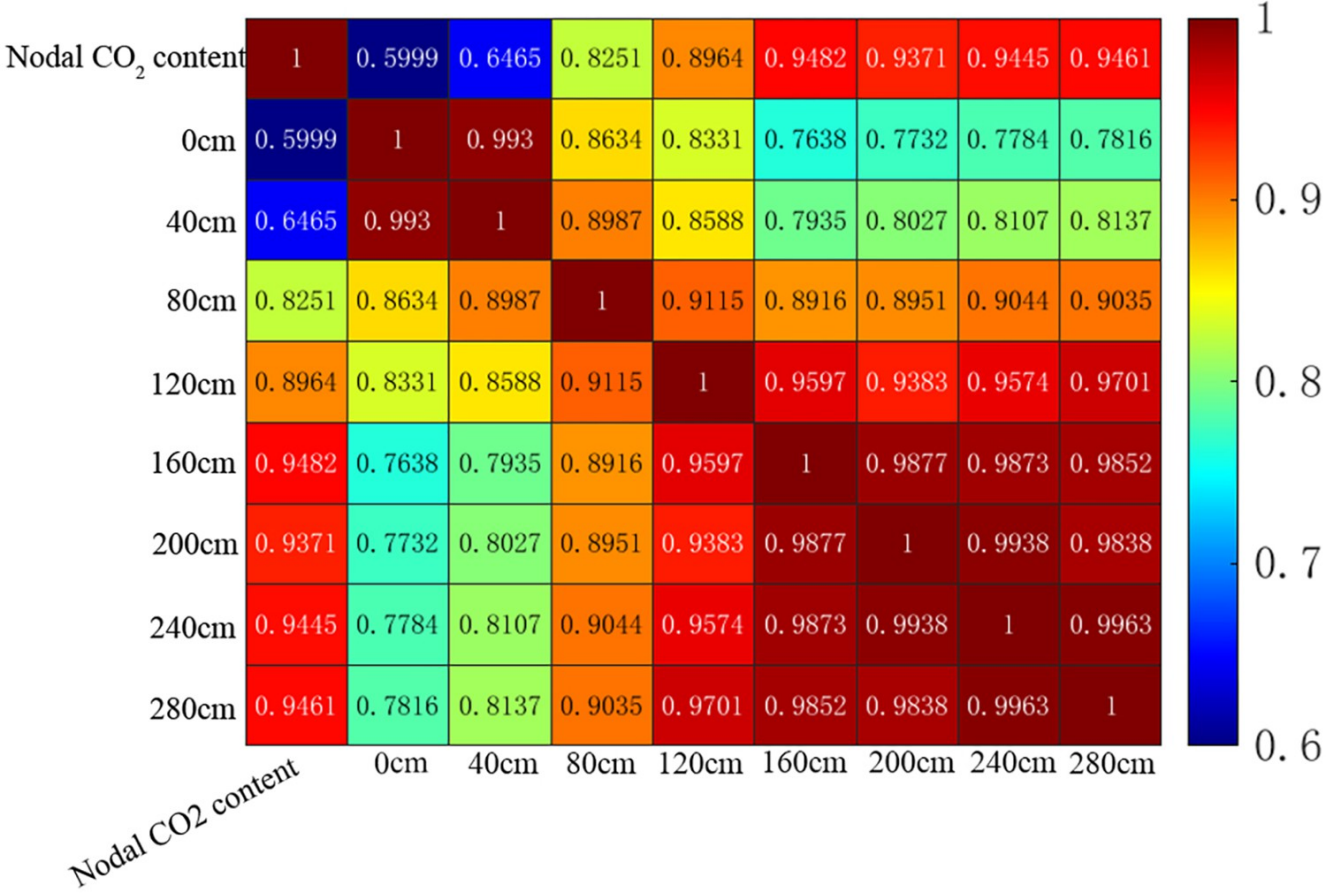
Heatmap of correlation coefficients between changes in CO2 concentration and temperature at the end section of the model.

The final section of the model in the similar fire experiment can be considered as the node of the ventilation network in an underground setting. In the event of a fire, the smoke reaching the final section of the model can be seen as the smoke spreading towards the ventilation network node, causing the fire hazard area to expand. The fire smoke consists of a significant amount of CO_2_ and CO, which are challenging to measure accurately due to the small fire and the low CO content produced by the combustibles in the similar fire experiment. To address this, we analyze the correlation by using data collected from CO_2_ concentration sensors at the final section of the model as the target sequence and comparing it with the output data from thermocouples in the tunnel. The heat map is shown in Fig 5.

**Fig 6:**
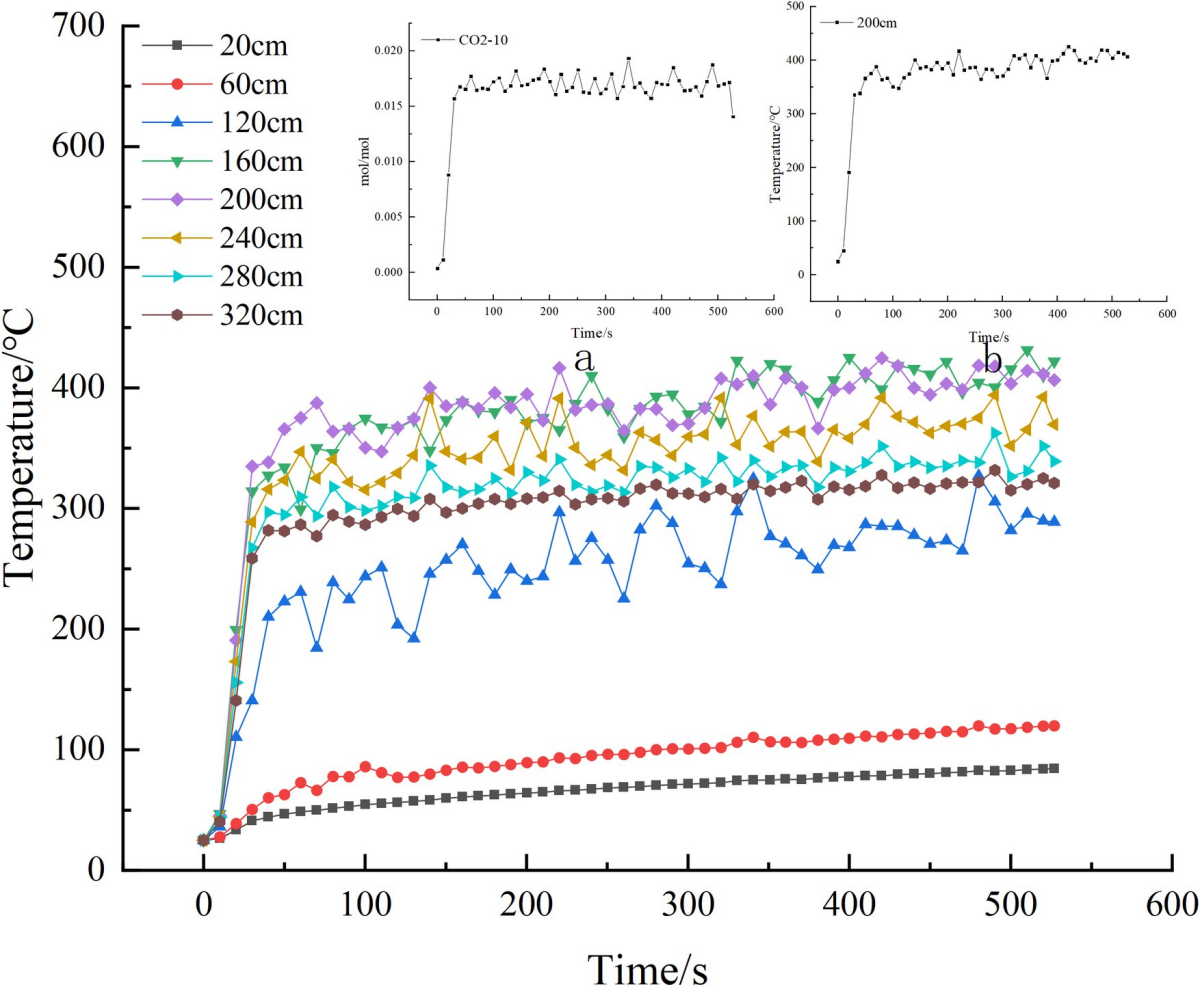
Plot of temperature change across thermocouples within the model (Figure a shows the change in CO_2_ concentration at the end of the model, Figure b shows the change in temperature at 200cm).

The closer the correlation coefficient is to 1, the higher the correlation is, and the more similar their geometries are, and the geometries of their target sequences with 200 cm are shown in Fig 6.

The heat map reveals a relationship between the thermocouple measurement point and the strength of the association with CO_2_. The correlation coefficients indicate that the highest degree of correlation is observed at a distance of 200cm from the source of the fire, with a correlation coefficient of 0.9482. Table 4 provides the correlation coefficients for different thermocouples and the end model of CO_2_.

**Table 4:** Temperature correlation of similar experiments.

order of relevance	Position of thermocouple from ignition source	relatedness
1	200 cm	0.9453
2	320 cm	0.9421
3	280 cm	0.9395
4	240 cm	0.9364
5	160 cm	0.8863
6	120 cm	0.8245
7	80 cm	0.6432
8	40 cm	0.5899

**Fig 7:**
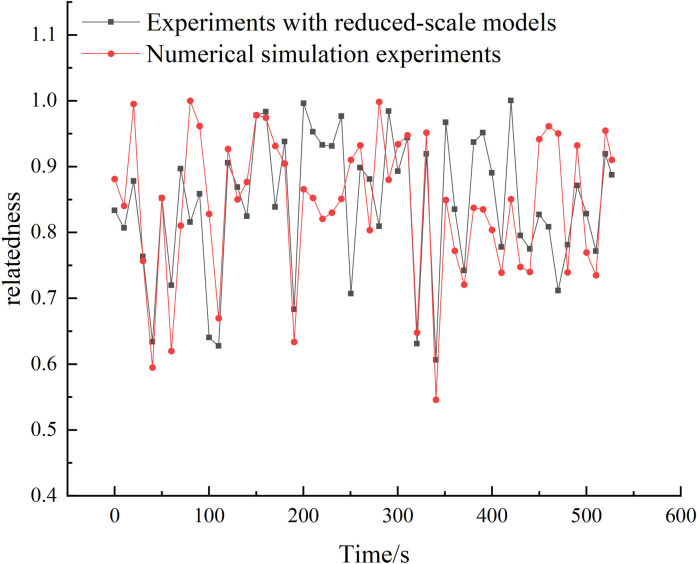
Correlation of CO_2_ at the same temperature measurement point with the end section for scale-down model experiments and numerical simulation experiments.

The optimized location for the fire alarm system is determined by selecting the location with the highest correlation between temperature change and CO_2_ concentration change. By comparing the correlation coefficients of the experimental conditions in a scaled-down model, it is found that the CO_2_ sensor at the end section of the model and the thermocouple placed at the downwind side of the fire source at a distance of 200cm can effectively detect fires by utilizing the correlation between CO_2_ concentration and temperature. Other thermocouples can be excluded from the alarm system, thereby optimizing the location and number of alarm sensors. The optimized correlation between thermocouples and CO_2_ exceeds 0.94, greatly enhancing the accuracy of fire detection. Additionally, a grey correlation analysis is conducted on the data obtained from FDS fire simulation, and the results are presented in Fig 7.

The graphical analysis reveals a strong correlation (0.91) between the grey correlation analysis results of the temperature measurement point and the terminal CO_2_ in both experiments. This indicates a high credibility of the numerical simulation.

The wind flow in the tunnel has an impact on the dispersion of high temperature smoke, with a stronger correlation observed closer to the end section of the model. However, the correlation coefficient does not solely depend on the distance and cannot be used as a criterion for determining actual tunnel fires. In addition to releasing high temperature smoke, mine fires also emit a significant amount of CO and affect the ventilation environment underground. To assess the correlation between fire characteristic gases, wind speeds, and ventilation nodes in the mine’s fire zone tunnels, a grey correlation analysis is conducted. Based on the results, a new sensor optimization scheme is proposed to enhance the accuracy and timeliness of the fire alarm system.

## 4 Sensor optimization position determination

### 4.1 Numerical simulation models

**Fig 8:**
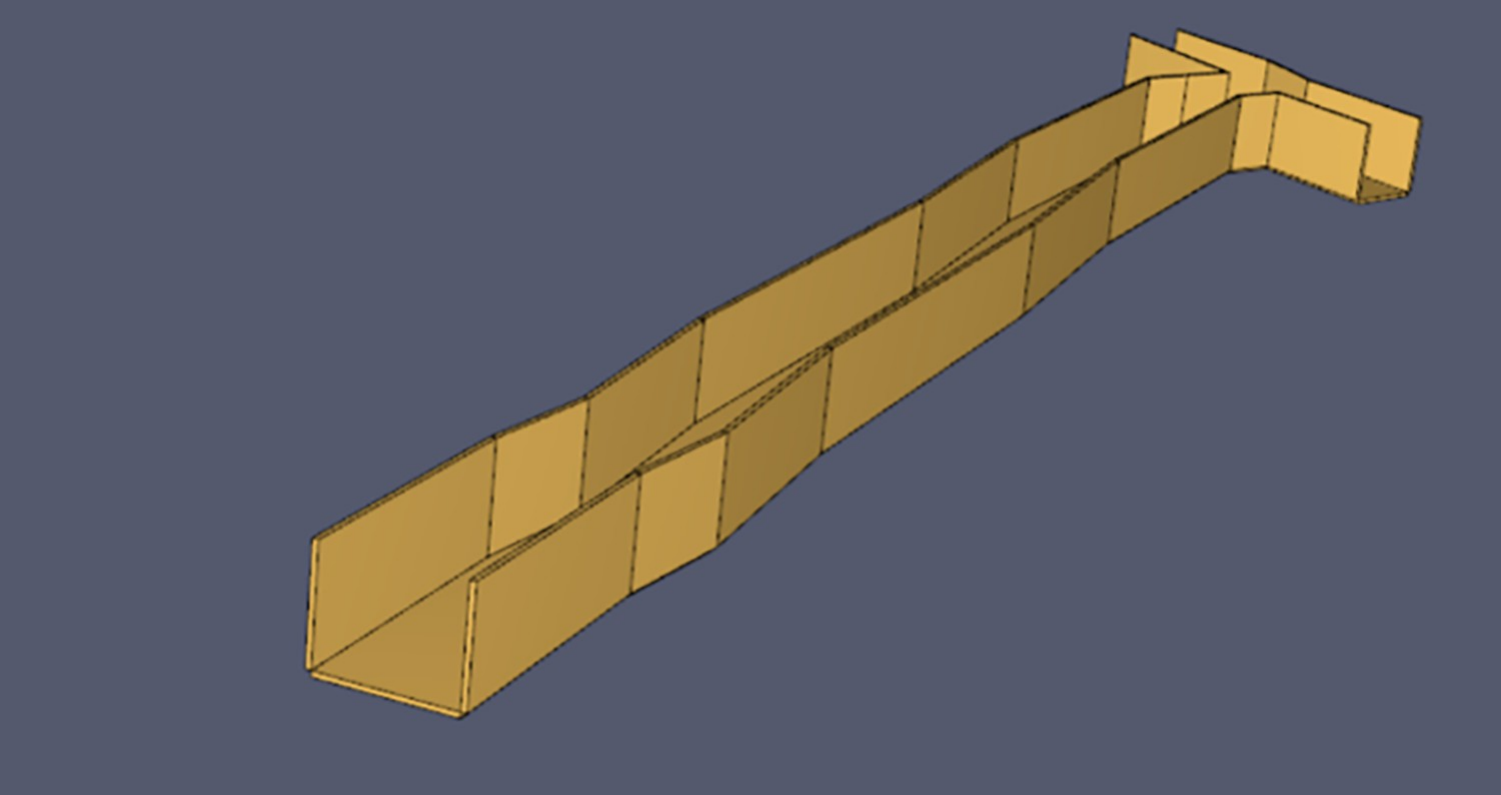
Full-size alleyway model drawing.

In order to better simulate the real environment under the mine, according to the relevant block diagrams, the FDS was used to build a tunnel with irregular walls. The tunnel is 200m long 5m wide and 4m high. The model diagram is shown in Fig 8.

The fire power is set to 5MW, and the t^2^ fire model is used, and its fire model can be described by the formula *Q = at*^*2*^. where *a* is the fire growth factor, *t* is the time for the fire power to reach the highest power, set to 263s, its fire growth factor is 0.072, the grid of two ports of the Y-type ventilation network is open, and the simulation time is 800s.

It has been shown that the accuracy of the simulation is higher when the grid size is about 16/1 to 1/4 of the characteristic diameter of the fire source [27].The FDS handbook proposes the parameter D*/& for the grid delineation, with D* being the characteristic diameter of the fire source, m; and & being the mesh size, m. It is calculated by the formula:

$$D*={\left(\frac{Q}{\rho_{a}c_{p}T_{a}\sqrt{g}}\right)}^{2/5}$$ (11)

Where *Q* is the power of the fire source, KW; *ρ*_*a*_ is the density of air, 1.205Kg/m^3^; *c*_*p*_ is the specific heat capacity of air at constant pressure, take 1.003KJ/(Kg·K); *T*_*a*_ is the ambient temperature, take 293K; *g* is the acceleration of gravity, take 9.81m/s^2^.

Eq 11 calculates D* = 2.3m, and the calculation of D*/*&* = 16/1~1/4 shows that the mesh size between 0.14m~0.57m is more accurate, and the mesh size of the numerical simulation is set to be 0.2m. Other numerical simulation boundary conditions are shown in Table 5.

**Table 5:** FDS full-size lane boundary conditions.

Boundary Condition	Parametric
Wall	CONCRETE
Roof and Floor	CONCRETE
Rection	Cellulose Combustion
Mesh Size	0.2m
Number of mesh areas	2
Number of mesh	502080

To analyze the correlation between carbon monoxide (CO) concentration, wind speed, and fire characteristics within tunnels, it is advisable to install wind speed sensors, CO sensors, and temperature sensors at ventilation nodes. These sensors should be mounted at a vertical height of 1.6 meters and positioned at intervals of 5 meters horizontally along the roof. Furthermore, CO sensors and wind speed sensor nodes should be specifically placed in the ventilation areas.

### 4.2 Analysis of numerical simulation experiments

Prior to the numerical simulation, the critical wind speed for the condition was first identified and Baker and Wu proposed a prediction model for the critical wind speed, which can be described as [28]:

$$\{\begin{array}{c} v^{*}=0.40{\left(0.2\right)}^{-1/3}Q^{*1/3}(Q^{*}\leq 0.20)\\ v^{*}=0.40(Q^{*}>0.20) \end{array}$$ (12)

$$v=v^{*}\cdot {\left(gH\right)}^{1/2}$$ (13)

where v* is the dimensionless wind speed; *v* is the critical wind speed, m/s; *H* is the hydraulic diameter, m; and Q* is the dimensionless heat release rate, which can be described by the equation [28]:

$$Q^{*}=\frac{Q}{\rho_{0}C_{P}T_{0}g^{1/2}H^{5/2}}$$ (14)

**Fig 9:**
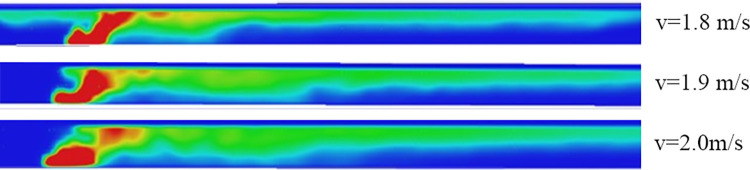
Comparison of temperature field under different wind speed conditions.

Where, *ρ*_*0*_ is the air density, Kg/m^3^; *T*_*0*_ is the air ambient temperature, K. The critical wind speed of 1.58m/s can be determined through calculation. In the test conditions, the wind speed was set at 1.58m/s as the midpoint, with incremental and decremental values of 0.1m/s on both sides. Several groups of experiments were conducted and it was found that the critical wind speed for this condition was 1.9m/s. In other ventilation environments with wind speeds less than 1.9m/s, varying degrees of smoke back occurred, which affected the escape environment on the upwind side of the fire source (Fig 9). This is due to the irregular shape of the wall and the friction on the wall surface which strengthens the resistance of the airflow along the flow. Therefore, in actual conditions, the critical wind speed is slightly higher than the calculated value.

**Fig 10:**
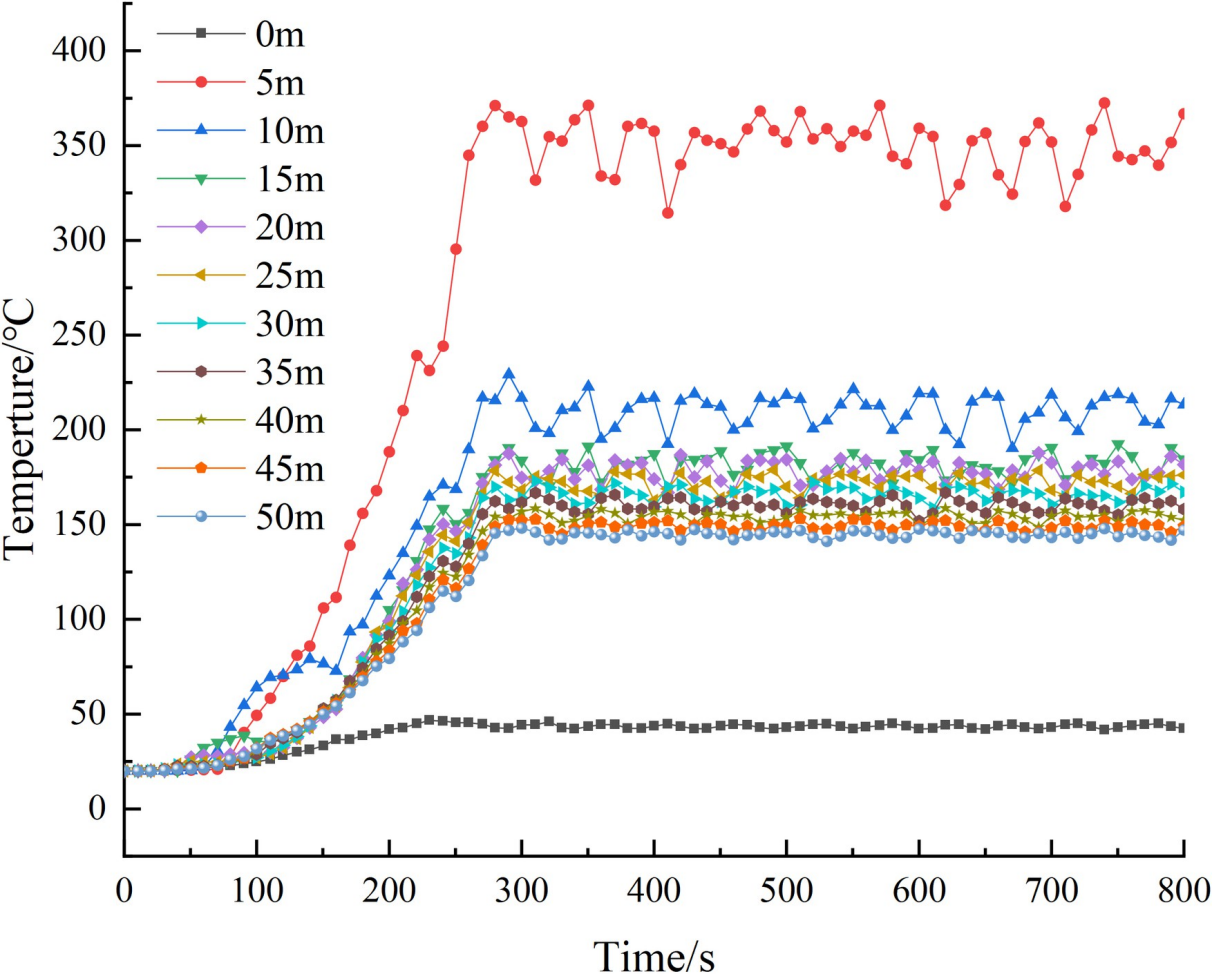
Tunnel roof temperature map.

In the simulation, it was observed that after approximately 300 seconds, the temperature in the tunnel reached a stable state with occasional fluctuations. The temperature near the fire source exhibited pulsation phenomena, leading to some variations. However, the wind speed directly above the fire source caused the flame front to deviate towards the downwind side. Compared to other monitoring points, the temperature change was relatively smaller. Fig 10 illustrates the temperature distribution on the tunnel roof.

**Fig 11:**
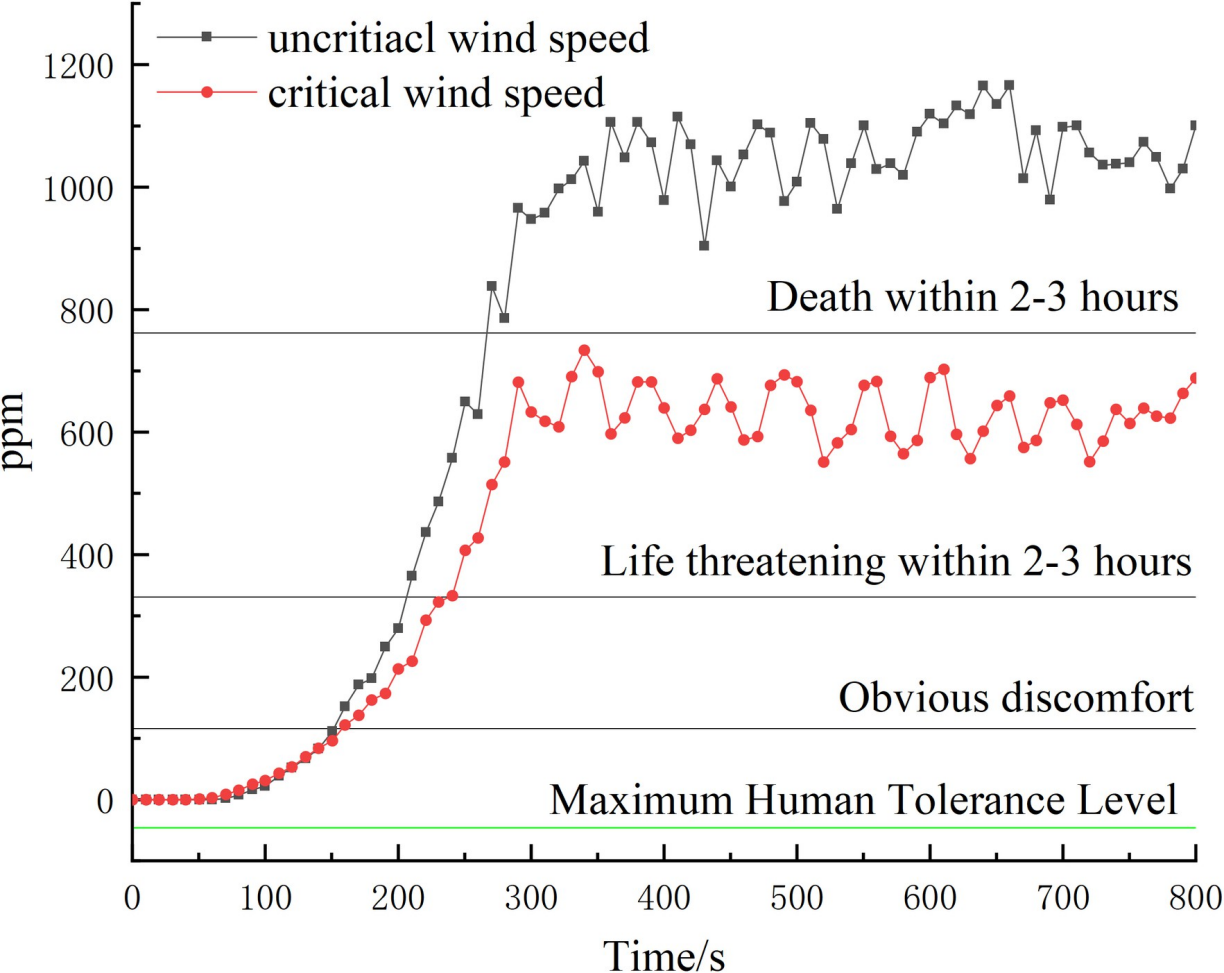
Plot of variation in CO content at ventilation nodes.

By examining the variation of CO content at the ventilation nodes under two different conditions, it is observed that the CO content in the condition with wind speed greater than the critical wind speed is considerably lower compared to the condition with wind speed below the critical wind speed. Furthermore, the CO content at the ventilation nodes in the condition with wind speed below the critical wind speed exhibits a more rapid trend of change. This information is visually represented in Fig 11.

CO content should be used as a key parameter to detect ventilation nodes. If excessive CO enters other mines through ventilation network nodes, it can quickly endanger tunnel personnel. The Table 6 shows the relevant indicators of CO that can harm the human body.

**Table 6:** Effects of different levels of CO on human health.

CO Content/ppm	Degree of harm
50	Maximum permissible level for adults
200	Headache,nausea,etc. in 2-3 hours
400	Life-threatening condition within 3 hours
800	Death with in 2-3 hours
1600	Death with in 1 hour

As depicted in Fig 11, the CO content in the ventilation nodes of both conditions reaches 50 ppm after 120 seconds of fire development. This level of CO poses a threat to the safety environment of other tunnels. Therefore, in the grey correlation analysis, it is important to determine the correlation coefficients between each fire characteristic parameter and wind speed within 120 seconds of the fire occurrence. This analysis ensures that the triggering alarms occur within 120 seconds, helping to prevent and control the enlargement of the disaster area.

### 4.3 Grey correlation analysis

Taking the change of CO content at the ventilation node and the change of wind speed as the target sequence, and the temperature, CO, and wind speed of each roof measurement point in the tunnel as the comparison sequence, grey correlation analysis was conducted to analyze the change of fire characteristic data of the two conditions within 120s, in which the grey correlation results of the CO content at the ventilation node and the fire characteristic data are shown in Table 7.

**Table 7:** Results of grey correlation analysis of CO with temperature and wind speed.

Comparsion Sequence	Critical Wind Speed Correlation	Non-Critical Wind Speed Correlation
Temperature directly above the fire source	0.725	0.823
Temperature 5m downwind of fire source	0.718	0.842
Temperature 10m downwind of fire source	0.732	0.835
Temperature 15m downwind of fire source	0.751	0.829
Temperature 20m downwind of fire source	0.745	0.829
Temperature 25m downwind of fire source	0.72	0.827
Temperature 30m downwind of fire source	0.719	0.828
Temperature 35m downwind of fire source	0.715	0.828
Temperature 40m downwind of fire source	0.716	0.827
Temperature 45m downwind of fire source	0.716	0.82
Temperature 50m downwind of fire source	0.717	0.82
Wind speed directly above the fire source	0.718	0.817
Wind speed 5m downwind of fire source	0.718	0.826
Wind speed 10m downwind of fire source	0.721	0.824
Wind speed 15m downwind of fire source	0.72	0.823
Wind speed 20m downwind of fire source	0.73	0.822
Wind speed 25m downwind of fire source	0.726	0.82
Wind speed 30m downwind of fire source	0.721	0.821
Wind speed 35m downwind of fire source	0.72	0.822
Wind speed 40m downwind of fire source	0.721	0.822
Wind speed 45m downwind of fire source	0.721	0.822
Wind speed 50m downwind of fire source	0.718	0.823

The results of the grey correlation between the ventilation node wind speed and fire characteristics data are shown in Table 8.

**Table 8:** Results of grey correlation analysis of wind speed with temperature and CO content.

Comparsion Sequence	Critical Wind Speed Correlation	Non-Critical Wind Speed Correlation
Temperature directly above the fire source	0.985	0.985
Temperature 5m downwind of fire source	0.985	0.984
Temperature 10m downwind of fire source	0.985	0.985
Temperature 15m downwind of fire source	0.976	0.984
Temperature 20m downwind of fire source	0.981	0.986
Temperature 25m downwind of fire source	0.975	0.986
Temperature 30m downwind of fire source	0.979	0.987
Temperature 35m downwind of fire source	0.981	0.984
Temperature 40m downwind of fire source	0.981	0.984
Temperature 45m downwind of fire source	0.981	0.987
Temperature 50m downwind of fire source	0.98	0.987
Wind speed directly above the fire source	0.944	0.912
Wind speed 5m downwind of fire source	0.941	0.927
Wind speed 10m downwind of fire source	0.944	0.95
Wind speed 15m downwind of fire source	0.965	0.958
Wind speed 20m downwind of fire source	0.955	0.951
Wind speed 25m downwind of fire source	0.941	0.949
Wind speed 30m downwind of fire source	0.972	0.94
Wind speed 35m downwind of fire source	0.969	0.939
Wind speed 40m downwind of fire source	0.971	0.935
Wind speed 45m downwind of fire source	0.969	0.93
Wind speed 50m downwind of fire source	0.94	0.929

The grey correlation analysis results showed that all the correlations between the nodal wind speed and the two working conditions were higher than 0.92, indicating a strong correlation. However, when the node CO content was used as the target sequence, the correlation degree varied. The highest correlation was observed between the wind speed greater than the critical wind speed and the temperature of 15m on the downwind side, with a correlation coefficient of 0.751. Although this correlation coefficient is not ideal, further analysis revealed that the correlation between the CO content at each point in the tunnel and the CO content at the ventilation node was excellent, with all correlations exceeding 0.94.

The correlation coefficient of CO content of ventilation node and the three factors of temperature, CO content and wind speed in the tunnel is calculated and ranked comprehensively, and it is *R*_*15*_>*R*_*20*_>*R*_*10*_>*R*_*50*_>*R*_*45*_>*R*_*25*_>*R*_*30*_>*R*_*40*_>*R*_*35*_>*R*_*0*_>*R*_*5*_. The correlation coefficients of CO content of ventilation node and the three factors of the fire in each point of the tunnel are averaged and calculated as a system correlation coefficient. The results are shown in Table 9.

**Table 9:** System correlation of each position sensor for different operating conditions.

Distance from ignition/m	System relevance	
	critical wind speed	uncritical wind speed
0	0.91	0.9
5	0.909	0.91
10	0.914	0.911
15	0.922	0.914
20	0.92	0.914
25	0.914	0.912
30	0.921	0.912
35	0.92	0.913
40	0.91	0.912
45	0.91	0.912
50	0.913	0.91

When the wind flow in the tunnel is greater than the critical wind speed, the system correlation coefficient is ranked as, *r*_*20*_ *= r*_*15*_*>r*_*35*_*>r*_*30*_ *= r*_*40*_ *= r*_*45*_ *= r*_*25*_*>r*_*10*_*>r*_*50*_ *= r*_*0*_*>r*_*5*_, and the system correlation is still the highest at 20m from the fire source when the wind flow is less than the critical wind speed. With the highest correlation coefficient as the optimization standard of sensor, therefore, fire alarm sensor should be set at 20m downwind side of the fire source and ventilation node, if the mine has the condition of setting more than one sensor, the distribution of its sensor should be in the relatively high correlation of the position of the priority of the sensor set up.

### 4.4 Fire alarm statute of limitations determination

To improve the accuracy of the alarm, it is important to confirm its response timeframe. This will help prevent the disaster from expanding and ensure the safe evacuation of people from the fire zone. Marchant outlines the conditions that need to be met for a successful evacuation, which can be found in reference [29]:

$$t_{p}+t_{a}+t_{rs}\leq t_{u}$$ (15)

**Fig 12:**
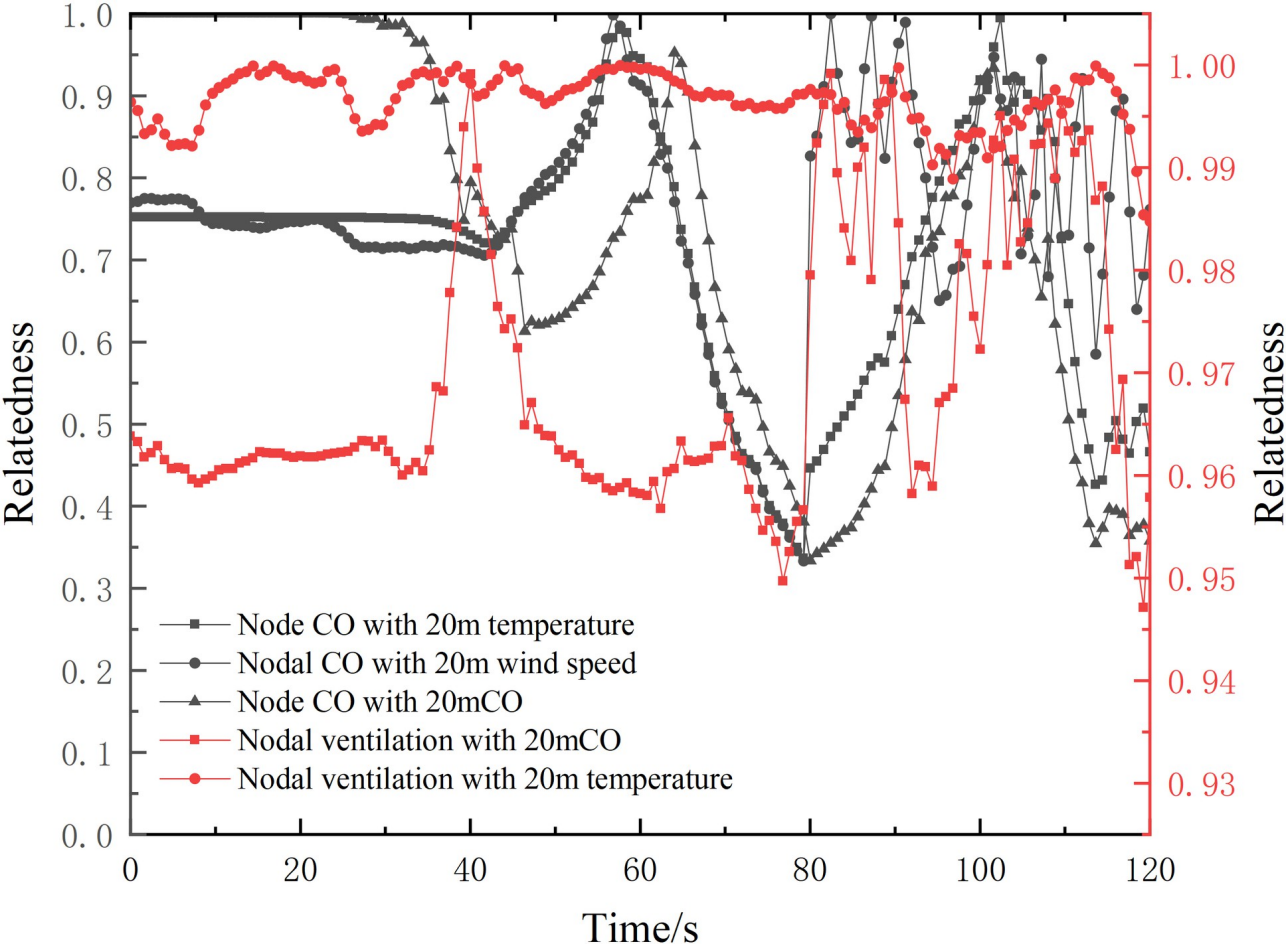
Change in correlation of factors over 120s.

Where *t*_*p*_ is the time elapsed from fire start to fire detection, s, which represents the optimized alarm response time; *t*_*a*_ is the time from fire detection to the start of evacuation activities, s; *t*_*rs*_ is the time from the time when the person enters the escape location, s; and *t*_*u*_ is the time from fire start to the time when the person is unable to tolerate the fire, s. If the wind speed in the fire lane exceeds the critical wind speed, it will have a lesser impact on the upstream escape of the fire. However, the downstream escape of the fire needs to be given more consideration. In this particular scenario, the fire source point is located 100m away from the ventilation node. The average escape speed for adults ranges from 2m/s to 5m/s. Assuming a personnel escape speed of 2.5m/s, it would take approximately 40s for individuals to evacuate the fire zone lane (*t*_*rs*_ = 40s). Research suggests that the personnel’s emergency response time is around 1.05s to 1.28s, and for the purpose of this study, ta can be set at 3s. It is worth noting that the CO content of the ventilation node can be correlated with time for the two aforementioned systems. For instance, at 120s, the CO content reaches about 50ppm (*t*_*u*_ = 120s), and the corresponding tp is 77s, as depicted in Fig 12.

According to the data presented in Fig 12, there is a noticeable decreasing trend in the correlation between CO levels at the ventilation node and CO levels 20m away from the fire source. This trend can be observed prior to approximately 44 seconds. Before 23 seconds, the correlation was at a value of 1. This can be attributed to the fact that during the initial stages of the fire, less CO is generated and the CO content gradually increases due to the influence of wind speed. As the fire develops, the CO content continues to rise. However, the propagation of CO to the ventilation node of the tunnel is limited, resulting in a rapid decrease in correlation. After 44 seconds, the correlation begins to increase once more, reaching its peak at approximately 60 seconds. This suggests that the correlation between the CO content at around 60 seconds and the three factors related to the fire in the tunnel is at its highest, thereby offering the greatest accuracy for the alarm system.

The highest correlation between the fire throttling effect and the wind speed at the ventilation node is observed at 44 seconds. Even at 60 seconds, the correlation remains high at 0.97. When the system is integrated for 60 seconds, the correlation reaches 0.94, indicating a strong relationship. Within the vertical direction of the 1.6m alleyway downstream of the fire, the fire characteristics parameters are shown below.

**Fig 13:**
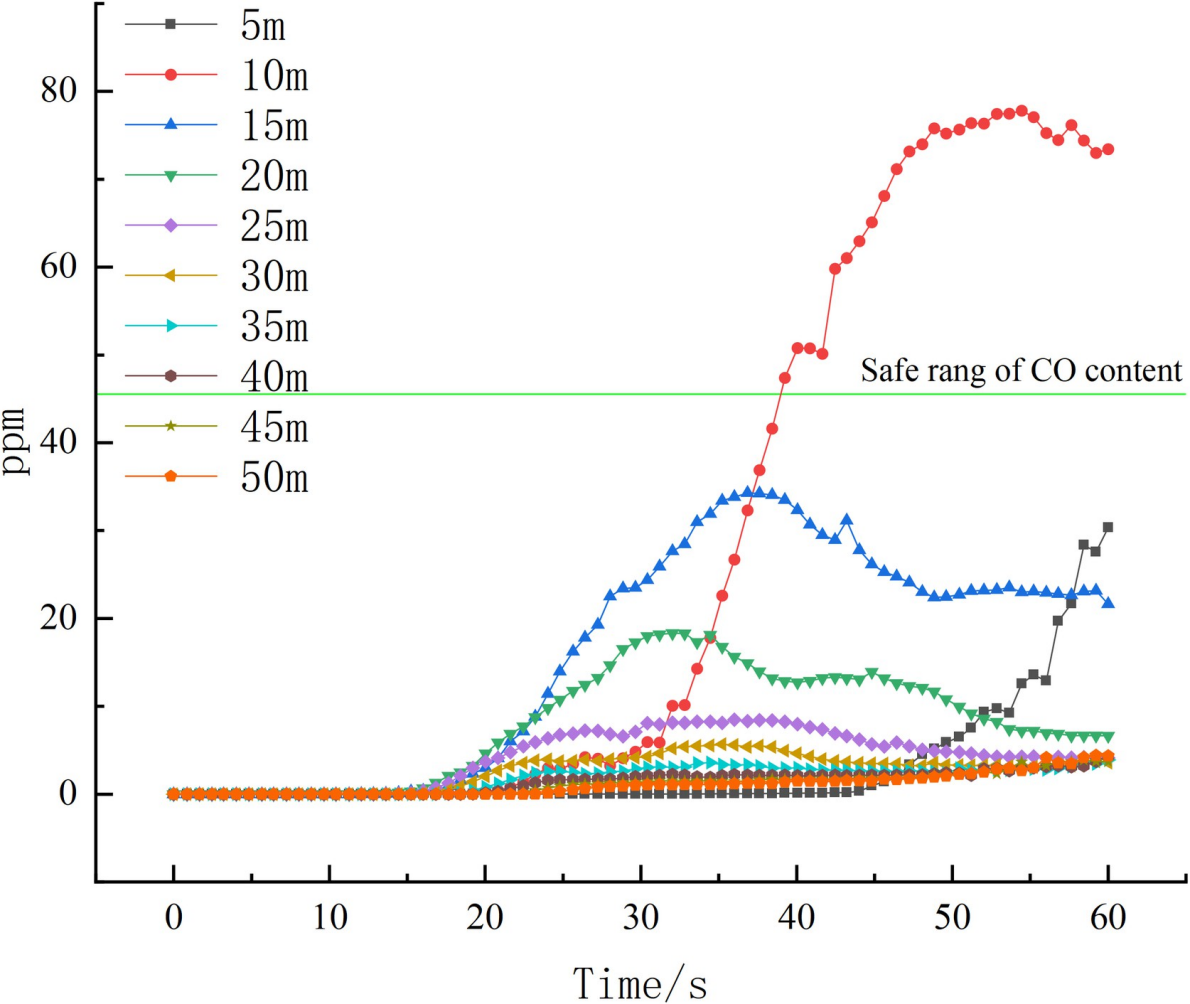
Change in vertical 1.6m CO content in 60s.

**Fig 14:**
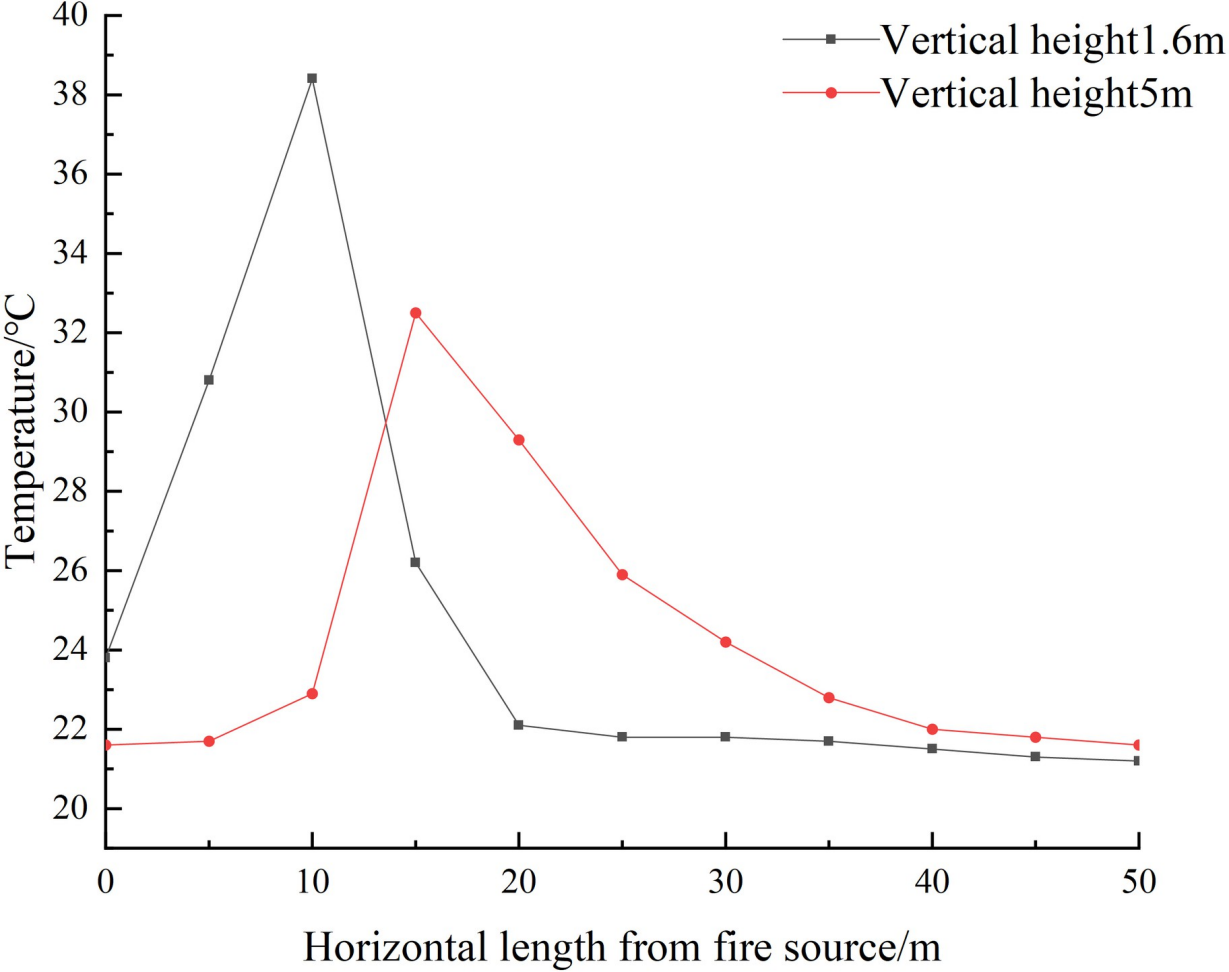
Temperature distribution at each vertical height at 60s.

In Fig 13, it is observed that the CO content at a distance of 10m from the fire source exceeds 50ppm. Additionally, Fig 14 reveals that at 60s after the fire starts, the maximum temperature recorded is 39°C. Although the escape of the people remains unaffected, it is worth noting that none of the measurement points reach the alarm threshold of the fire sensor.

In this study, grey correlation analysis was employed to investigate the relationship between changes in wind speed and CO levels at ventilation nodes. The objective was to optimize both the location and type of information collected for multi-factor fire alarms within tunnels. By correlating multiple factors, the accuracy and timeliness of fire alarms could be improved. Additionally, if a fire alarm is triggered, the ventilation node can be promptly blocked using a damper, and a disaster response plan can be implemented in advance to prevent the escalation of the situation.

## 5 Conclusion

1) The correlation analysis between the CO2 content and the internal temperature of the model was conducted using grey correlation analysis. Similar experiments and numerical simulations were performed to determine the location with the highest correlation, which was found to be 200 cm away from the fire source, with a correlation coefficient of 0.9482. The results of the experiments and simulations were highly consistent, indicating a high degree of similarity. The grey correlation analysis method used in the numerical simulations provided more credible results;

2) The full-size tunnel fire was simulated using the numerical simulation software FDS. Through the experiment, it was determined that the critical time for the spread of CO leading to the expansion of the disaster area is 120s. Fire characteristic parameters of the experimental conditions were subjected to grey correlation analysis at different wind speeds. The results revealed that the fire characteristic data collected at a distance of 20m from the source of the fire had the highest systematic correlation, regardless of the wind speed of the tunnel being critical or not;

3) The sensor optimization scheme for experimental conditions with different wind speeds is provided. The sensors should be placed in locations with high system correlation. The experimental conditions should be installed at a distance of 20m from the fire source and the ventilation node. It has been determined that the optimized scheme can trigger the alarm within 60s. In this optimized scheme, the system correlation is greater than the critical wind speed at 0.922, and it is 0.914 for wind speeds below the critical value. The escape environment for personnel is excellent before and after the alarm;

4) The installation of sensors in the tunnel should consider flammable materials as the reference point for detecting fire sources. This method of sensor optimization can reduce the number of sensors required and provide specific distribution positions for them. Additionally, it can serve as a reference for the development of a multi-factor fire alarm platform, improving the accuracy of fire alarm systems and preventing the escalation of disasters.

## Supporting information

S1 File Supporting information can be found in compact package supporting information. (7Z)
